# News and Notes

**Published:** 1906-03

**Authors:** 


### *NEWS AND NOTES.*

---

AN anticigarette bill similar to that passed in Indiana is soon to be introduced into the Ohio legislature.

---

F. M. BRUCE, M.D., Pineview, Ga., was married to Miss Annie Pearl Mitchell, of Abbeville, Ga., January 7th.

---

NORWAY, it is claimed, forbids the newspapers to publish advertisements or in any other way to further the sale of any and all patent medicines.

---

THE Virginia Senate has passed the House bill providing for the establishment of a home for epileptics. The hospital will be located near Lynchburg.

---

GEORGE W. T. HANNAH, M.D., Vanderbilt University Medical Department, Nashville, Tenn., 1876, died suddenly at his home in Thomaston, Ga., February 1st.

---

J. HOWARD HUGHES, a senior medical student at the University of Maryland, was killed by the accidental discharge of his pistol at Jersey City, N. J., February 4th.

---

A COMMITTEE has been formed under the patronage of Prince Bernhard of Saxe-Meiningen for the erection of a memorial to the late Professor von Mikulicz at Breslau.

---

THE Berlin ambulance and first-aid stations gave first aid during the last year in 54,678 cases, twenty stations being made use of, and 1,980 times ice was given in emergencies.

DR. GEORGE W. WENDE, of Buffalo, was elected secretary and treasurer of the American Dermatological Association at its recent annual meeting held in New York, December 28, 1905.

---

DR. WILLIAM RANEY HARPER, president of the University of Chicago, and one of the world's greatest authorities on Hebrew and Sanscrit, died at his home in Chicago, January 10, 1906.

---

AT Meridian, Miss., on January 4th, two men giving their residence as Ohio were tried before Justice Townsend for selling patent medicines without a license and were fined $50 and costs.

---

IN a report from Colonel Gorgas, he states that Panama has been entirely free from yellow fever for more than two months, and that the health, in general, on the isthmus is said to be excellent.

---

PATENT MEDICINE PEDDLERS who go about the country vending preparations containing alcohol must hereafter pay a special tax at every separate place where they make any sales. This is the order issued by Commissioner Yerkes to all collectors of internal revenue.

---

DR. JOHN WILLIAM BALLENTYNE, of Edinburgh, Scotland, has been appointed professor of midwifery and diseases of women and children in the University of Edinburgh. Professor Ballentyne is an Honorary Fellow of the American Association of Obstetricians and Gynecologists.

---

ON Friday, January 26th, the members of the Medico-Psychological Association presented Dr. Henry M. Hurd, superintendent of Johns Hopkins Hospital, a handsome loving-cup in appreciation of his long and faithful service in the affairs of the association. Dr. Charles G. Hill made the presentation address.

MEMPHIS is to have a new medical college to be known as the College of Physicians and Surgeons. The land, which has already been purchased, is 115 feet by 333 feet. Dr. Heber Jones, president of the Memphis Board of Health, is to be president of the new institution and dean of the faculty.

---

THE Japanese Minister of War says that Japan at one time in the course of the war had 1,200,000 troops under arms. Of this number, said the minister, 70,000 died and 310,000 were wounded or became sick, but only 15,000 died from sickness and 9,800 from wounds after coming under treatment.

---

A COLLECTION of rare old books and pamphlets, once the property of Professor Ahlfeld, of Marburg, Germany, has been presented to the Johns Hopkins University library, by Mr. F. M. Jenks, a member of the board of trustees of Johns Hopkins Hospital. They number 934, and are said to be very valuable.

---

IT is announced that all first-class passengers landing at American ports are in future to be subjected to a medical examination as rigorous as is enforced in the case of steerage passengers. Up to the present the first-class passengers have escaped the medical inspector, who has accepted the statement of the ship's doctor as to their health.

---

TWENTY-EIGHT young women received diplomas from the Bellevue Training School, New York, on January 9th. The Board of Estimate decided that the new home nurses should be provided for by the city, therefore the $50,000 recently collected for that purpose will be used for the running expenses of the home.

---

BEGINNING NEW BELLEVUE.—Two brick pavilions on the southeast corner of the grounds have been emptied of patients and will be razed. This is the first step in the erection of the new hospital for which $8,500,000 has been appropriated. The consumptive patients have been sent to the Metropolitan Hospital while the others will be cared for in tents.

---

A BILL recently was introduced in the Maryland House and Senate to secure an appropriation of $50,000 annually for the years 1906 and 1907 for the erection of an extension to the University Hospital on West Lombard Street. The medical faculty of the University of Maryland will raise for this purpose $200,000 and asks the State to furnish the balance. Next year the University will complete a century of its existence, and the bill is said to be strongly endorsed by the medical profession throughout Maryland.

---

THE LOUISIANA STATE BOARD OF HEALTH was reorganized with the following membership on January 8th: President, Dr. C. H. Irion, New Orleans; Vice-President, Dr. W. G. Owen, White Castle; members, Dr. G. W. Gaines, Tallulah; Dr. A. J. Perkins, Lake Charles; Dr. T. E. Schumpert, Shreveport; Dr. J. M. Batchelor, New Orleans; Dr. W. G. Armstrong, New Orleans, and W. S. Ingram, secretary, New Orleans. The central office of the Board will continue at 204 Carondelet street, New Orleans, La.

---

THE McDUFFIE COUNTY MEDICAL SOCIETY was organized at Thomson, Ga., on Wednesday, January 17th, under the constitution and by-laws of the Medical Association of Georgia. The following officers were elected: President, Dr. E. S. Harrison, Thomson; vice-president, Dr. Sterling Gibson, Thomson; secretary and treasurer, Dr. B. F. Riley, Jr., Thomson; board of censors, Dr. F. N. Ware and Dr. A. J. Matthews, Thomson, and Dr. Z. D. Story, Winfield; delegate to the legislative council of the Georgia Medical Association, Dr. Z. D. Story.

F. SCHAUDINN of Berlin has been working lately in the Hamburg Institute for Tropical and Ship Diseases, under commission from the Imperial Council of Health. The Hamburg authorities have now decided to invite him to become permanently connected with the institution, the official title of which is the "Institut für Schiffs- und Tropenkrankheiten." He is to be assistant in zoology with a salary of 9,000 marks (about $2,250), and a pension later. The sum of $14,250 has been appropriated to enlarge the institute. Schaudinn's fame as an authority on protozoa was so well established that when he announced the discovery of the *Spirochæta pallida* the world stopped at once to listen, while the words of a lesser authority might not have attracted attention for some time.—*Jour. A. M. A., Feb. 17, '06.*

---

A MOVEMENT for the arrangement of a Sunday rest for medical practitioners has been set on foot in Germany. So far, the plan has come in operation only in one place—Freising. In that happy town it has been arranged that every Sunday from noon till midnight two practitioners shall alternately undertake to attend to all new calls. An intimation of the practitioner on duty is made by notices published in the papers and by boards affixed to the doctor's dwelling and hung up in druggists' shops. The plan is said to have been well received by the press and the public. A similar system has, it is said, been adopted at Frankfort. The city has been divided into seventeen districts, corresponding to the police division, and the practitioners in each district have agreed to take their turns in being on duty for their neighbors on Sundays and holidays.—*British Med. Jour., February 3, '06.*

---

THE Rockefeller Institute for Medical Research offers for 1906-07 scholarships to assist investigations in experimental pathology, bacteriology, medical zoology, physiology and pharmacology, and physiologic and pathologic chemistry, to be carried on in the laboratories in the institute in New York City. The value of these scholarships ranges from $600 to $1,000. They are open to men and women who are properly qualified to undertake research work in any of these subjects and who will devote their entire time to it. Applications and credentials should be in the hands of the secretary of the Rockefeller Institute, Dr. L. Emmet Holt, 14 West Fifty-fifth Street, New York City, not later than April 1, 1906. The announcements of the appointments is made about May 15th. The term of service begins preferably on October 1st, but, by special arrangement, may be begun at another time.—*Jour. A. M. A., February 10, '06.*

---

THE PASSING OF THE FIJIANS.—The *British Medical Journal* states that the total population of the Fiji Islands is 121,773, according to the Colonial Office report, an increase of about 2,000 on the numbers of the last census in March, 1901. Of these, the pure-blood Fijians number 90,063, from which it appears that there has been the lamentable decrease in this short period of three years and a half of no less than 4,334. Most of this was due to a severe epidemic of measles in 1903, in which year the natives decreased by 2,481. In 1904 the decrease was 840, and it would seem as if the native race has been slowly but steadily disappearing ever since the terrible epidemic of measles in 1875, when over 40,000 are believed to have perished. The native death-rate was 48.90 per mille last year, as against a white death-rate of 15.25, and this with no epidemics and with a birth-rate of nearly 40. The report does not comment on this high mortality which has been so long existent, but something might surely be done to ameliorate such conditions, though "native superstition and obstinacy" are represented as militating considerably against the measures lately taken to stamp out yaws.—*Jour. A. M. A.*

